# Dynamics of Cdk1 Substrate Specificity during the Cell Cycle

**DOI:** 10.1016/j.molcel.2011.05.016

**Published:** 2011-06-10

**Authors:** Mardo Kõivomägi, Ervin Valk, Rainis Venta, Anna Iofik, Martin Lepiku, David O. Morgan, Mart Loog

**Affiliations:** 1 Institute of Technology, University of Tartu, Tartu 50411, Estonia; 2 Department of Physiology, University of California, San Francisco, San Francisco, CA 94158, USA

## Abstract

Cdk specificity is determined by the intrinsic selectivity of the active site and by substrate docking sites on the cyclin subunit. There is a long-standing debate about the relative importance of these factors in the timing of Cdk1 substrate phosphorylation. We analyzed major budding yeast cyclins (the G1/S-cyclin Cln2, S-cyclin Clb5, G2/M-cyclin Clb3, and M-cyclin Clb2) and found that the activity of Cdk1 toward the consensus motif increased gradually in the sequence Cln2-Clb5-Clb3-Clb2, in parallel with cell cycle progression. Further, we identified a docking element that compensates for the weak intrinsic specificity of Cln2 toward G1-specific targets. In addition, Cln2-Cdk1 showed distinct consensus site specificity, suggesting that cyclins do not merely activate Cdk1 but also modulate its active-site specificity. Finally, we identified several Cln2-, Clb3-, and Clb2-specific Cdk1 targets. We propose that robust timing and ordering of cell cycle events depend on gradual changes in the substrate specificity of Cdk1.

## Highlights

► Cyclins gradually raise the active-site specificity of Cdk1 during the cell cycle ► A novel substrate docking motif is specific for G1-cyclin-Cdk1 complexes ► G1 cyclin-Cdk1 shows distinct features of substrate site consensus specificity ► Several G1, G2, and M phase-specific Cdk1 targets exist in the cell

## Introduction

The rise or fall of different cyclin levels is an important switching mechanism triggering the major events of the cell division cycle. The three major switch points that are controlled by cyclin-Cdk activity can be distinguished as Start (G1/S), mitotic entry, and the metaphase-anaphase transition (Morgan, 2007). The general principle of sequential cyclin signals as a periodic driving force of the cell cycle is conserved throughout the eukaryotes. In the budding yeast *Saccharomyces cerevisiae*, cyclins Cln1–3 are triggers for G1 and G1/S, Clb5 and Clb6 drive S phase, Clb3 and Clb4 are specific for early mitotic events, and Clb1 and Clb2 complete the progression to mitosis. A single Cdk, Cdk1, associates with these cyclins to mediate all major cell cycle transitions. However, despite extensive studies in multiple model organisms, there is still no general model of the functional specificity of cyclins, which would explain how they provide temporal separation for cell cycle transitions.

In the course of the cell cycle, cyclins appear and disappear as periodic and partly overlapping waves. Quantitative analysis of cycling cultures of budding yeast suggests that the abundance of different cyclins is relatively similar, with only a few-fold variance (Cross et al., 2002). This finding suggests that one can consider the period from G1 to mitotic exit as a state of relatively unchanging net levels of activated Cdk1. Therefore, it is unlikely that thresholds of total cyclin levels are responsible for triggering cell cycle transitions; instead, it is more likely that precise control of cell cycle transitions depends on differential substrate recognition by different cyclin-Cdk complexes. Cyclin-dependent substrate specificity of Cdk1 has been addressed in several previous studies (Cross et al., 1999; Ikui et al., 2007; Loog and Morgan, 2005), but the general dynamics of Cdk specificity in the cell cycle remain unclear. If differences in target recognition among the cyclin-Cdk complexes exist, then to what extent are these complexes able to provide exclusively specific signals, and to what extent is the specificity overlapping? Clearly, some mechanism must exist that, in addition to providing high specificity toward some substrates, also eliminates activity toward substrates associated with other cell cycle stages. Why, for example, does the activation of Cdk1 in the early cell cycle by G1/S cyclins not trigger premature S or M phase processes? On the other hand, there should exist a common specificity among different complexes toward some Cdk targets. To prevent rereplication, for example, components of the prereplicative complex should be kept in their phosphorylated state throughout the cycle by all B-type cyclin-Cdk1 complexes (Nguyen et al., 2001).

In vivo evidence hints that a filtering mechanism might prevent early cyclins from acting prematurely on later triggers. A number of experiments with cultured cells and transgenic mice suggest that some mammalian cyclins can functionally compensate for the absence of others. However, of all the different cyclins tested, the later mitotic cyclins A2 and B1 appeared to be the most nonredundant, suggesting that the early cyclins cannot perform their function (Satyanarayana and Kaldis, 2009). Genetic evidence from budding yeast has indicated that the major cyclin genes are not fully interchangeable, as replacement of the budding yeast S phase cyclin Clb5 with the mitotic cyclin Clb2 causes replication defects (Cross et al., 1999; Cross and Jacobson, 2000; Donaldson, 2000). Similarly, early cyclins appear less capable of performing later Cdk functions, as the deletion of both Clb5 and Clb6 results only in an S phase delay (Schwob and Nasmyth, 1993), whereas the deletion of both mitotic cyclins is lethal (Mendenhall and Hodge, 1998). In fission yeast, early cyclins can be deleted without severe consequences, while the mitotic cyclin is essential for division (Fisher and Nurse, 1996).

Our goal in the present study was to analyze the dynamics of Cdk1 specificity during the cell cycle of budding yeast. Operating with a single Cdk1 molecule makes the yeast cell cycle an ideal model for comparative studies of the differential impact of cyclins on Cdk function. We carried out a detailed specificity study of the four major classes of cyclin-Cdk1 complexes and outlined the general mechanisms underlying changes in Cdk1 specificity during the cell cycle. We propose that the later appearing cyclins gradually increase the specificity of Cdk1 toward mitotic targets by improving its ability to recognize the optimal Cdk consensus site. On the other hand, at the early stages of the cell cycle, Cdk1 is able to target selected substrates using docking sites and cyclin-specific consensus motifs. This model explains the paradox raised by the in vivo studies referred to above, answering the question of why the early cyclins are incapable of triggering prematurely the later stages of the cycle. Also, quite surprisingly, we found that cyclins may differentially modulate the optimal consensus motif of Cdk1, revealing a previously unappreciated cyclin function. Our results suggest that the robust ordering of cell cycle events depends on gradual changes in cyclin specificity.

## Results

### The Cyclins Gradually Change the Specificity of Cdk1

**Figure 1:**
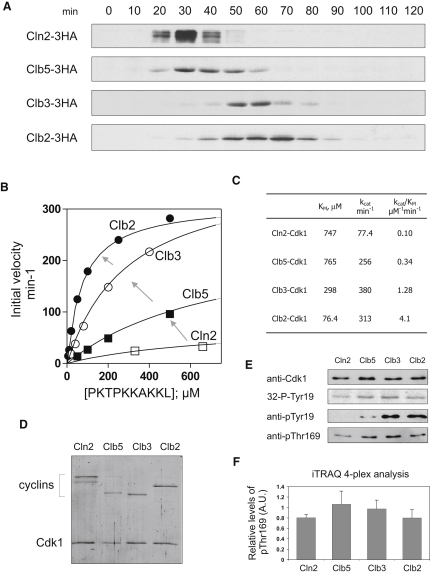
Intrinsic Specificity of Cyclin-Cdk1 Complexes toward the Histone H1-Based Model Substrate PKTPKKAKKL Increases Gradually in the Series Cln2-Clb5-Clb3-Clb2, Correlating with Cell Cycle Progression (A) Western blotting analyses of endogeneous cyclin levels in the course of the cell cycle. The time points were taken after the release of cells from α factor-induced G1 arrest. The strains carried the indicated cyclin with a C-terminal 3HA tag. (B) Michealis-Menten curves of Histone H1 peptide (PKTPKKAKKL) phosphorylation by four different cyclin-Cdk1 complexes. Arrows indicate the change of Cdk1 kinetic properties during cell cycle progression. (C) Steady-state kinetic parameters determined for four representative cyclin-Cdk1 complexes using Histone H1-based peptide PKTPKKAKKL as a substrate. (D) Silver-stained SDS gel showing the purified preparations of cyclin-Cdk1 complexes used in this study. (E) Analyses of the levels of activating phosphorylation at T169 and inhibitory phosphorylation at Y19 of cyclin-Cdk1 preparations. Equal protein amounts of each cyclin-Cdk1 preparation were loaded and blotted using specific antibody for Cdk1 (upper panel). The second panel shows the active site titration of cyclin-Cdk1 preparations using an excess of purified Swe1. Equal protein amounts of cyclin-Cdk1 preparations were phosphorylated with Swe1 until Cdk1 was totally inactivated. The third panel shows the relative levels of pY19 initially present in the preparations, determined using western blotting with specific antibody. The lower panel shows western blotting to determine the relative levels of activating phosphorylation at T169 (see the Supplemental Experimental Procedures for details). (F) iTRAQ four-plex analysis of the relative levels of activating phosphorylation at T169 in cyclin-Cdk1 preparations. Cyclin/Cdk1 complexes were separated by 10% PAGE, and the Cdk1 band was excised and digested with trypsin. Eluted peptides were labeled with iTRAQ reagents and analyzed by tandem mass-spectrometry (see the Supplemental Experimental Procedures for details). Intensities of four reporter ions at m/z 114, 115, 116, and 117 are presented. Analyses were performed in triplicate, and error bars indicate standard error of the mean.

To study how different cyclins modulate the substrate specificity of Cdk1, we performed a quantitative analysis of yeast Cdk1 specificity in complex with each of four cyclins: the G1/S cyclin Cln2, the S phase triggering cyclin Clb5, the G2/M cyclin Clb3, and the mitotic cyclin Clb2. The cellular levels of these four cyclins peak in sequence over the course of the cell cycle (Figure 1A). We purified the four cyclin-Cdk1 complexes from yeast (Figure 1D) and analyzed their kinetic properties with a commonly used optimal model substrate for Cdk1, a peptide derived from the general substrate Histone H1 (PKTPKKAKKL). We showed previously that this substrate has about 20-fold higher specificity for the mitotic Clb2-Cdk1 complex as compared with the S phase Clb5-Cdk1 complex (Loog and Morgan, 2005). We extended this work in the current study, finding that each of the four major cyclin-Cdk1 complexes exhibited different specificity toward this substrate (Figures 1B and 1C). Remarkably, these specificity differences were gradual and correlated with the order of appearance of the cyclins in the cell cycle. This difference was mostly manifested in K_M_ values, while the k_cat_ values showed much smaller variation (Figure 1C).

Next, we asked if this gradually changing ability to phosphorylate the substrate could be due to differences in posttranslational modifications of Cdk1. We analyzed two known regulatory phosphorylation sites of Cdk1: the inhibitory site at Y19 and the activating site at T169. First, we performed an active site titration by determining the concentration of unphosphorylated Y19 sites in our enzyme preparations, using an excess of purified Swe1 to radiolabel these sites (Figure 1E). The amount of Y19 labeling was similar for the four cyclin-Cdk1 complexes. Next, we estimated the relative levels of inhibitory phosphorylation already present in the enzyme preparations. Western blotting analysis revealed that pY19 levels increased gradually in the cyclin series, being lowest in the case of Cln2-Cdk1 and highest in the case of Clb2-Cdk1. These data are in agreement with previously reported observations that Cln2-Cdk1 is a poor substrate for Swe1 (Booher et al., 1993), and that Clb5-Cdk1 is less susceptible than Clb2-Cdk1 to inhibition by Swe1 (Hu and Aparicio, 2005; Keaton et al., 2007). However, the different levels of inhibitory phosphorylation at Y19 cannot be responsible for the activity differences observed for individual cyclin-Cdk1 complexes, since these changes occur in the opposite direction. Thus, differences in the western blotting experiment represent a trace fraction of the total enzyme in each case.

Using specific antibodies against the phosphorylation site pT169, we found that activating phosphorylation was present at equal levels in all Cdk1 preparations tested (Figure 1E). To confirm this result, we used a quantitative mass-spectrometry-based iTRAQ four-plex analysis of pT169 levels in the four cyclin-Cdk1 preparations (see the Supplemental Experimental Procedures, available with this article online, for details). This technique indicated that the levels of pT169 were equal for the four kinase complexes (Figure 1F).

### Cyclin-Specific Docking Sites Are Able to Compensate for the Gradually Decreasing Specificity of Early Cyclin-Cdk1 Complexes

Our data reveal that cyclins are not just activators of Cdk1 but are also modulators of the catalytic specificity of the kinase active site. This gradual increase in the intrinsic activity toward the optimal substrate motif in the course of the cell cycle could provide an important delay in the accumulation of mitosis-promoting activity, preventing the premature initiation of mitotic processes by Cdk1. On the other hand, the fact that the early cyclin-Cdk1 complexes have very low intrinsic activity raises the question of how these complexes initiate such important events as Start and S phase. In our previous studies, we found that Clb5-Cdk1 can compensate for its low intrinsic activity by using a docking site on its cyclin surface—the hydrophobic patch—that binds selectively to an R/KXL motif in the substrate (Loog and Morgan, 2005). In contrast, the intrinsically potent Clb2-Cdk1 does not seem to utilize extra specificity from this docking site.

**Figure 2:**
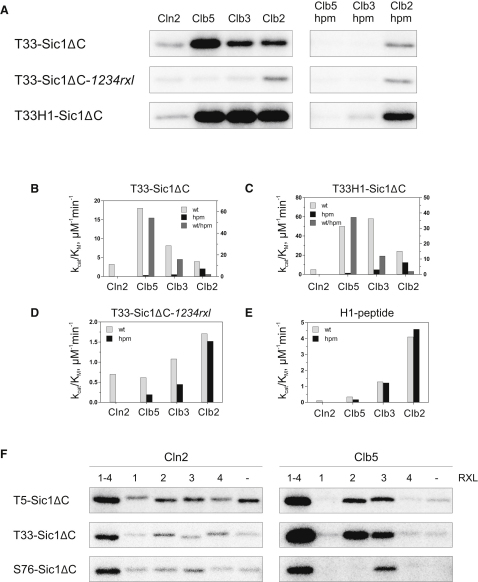
Analysis of Cyclin Specificity with Respect to RXL Docking Sites (A) Equal amounts of cyclin-Cdk1 complexes were used in a phosphorylation assay with purified T33-Sic1ΔC, T33-Sic1ΔC-*1234rxl*, and T33H1-Sic1ΔC. The first two mutant proteins contained the original sequence of T33, QA^33^TPQKPSQNL, while the last contained the Histone H1 peptide sequence PK^33^TPKKAKKL in place of the T33 site of T33-Sic1ΔC. (B–D) Quantified specificity profiles showing the k_cat_/K_M_ values obtained from the experiments shown in (A), using the indicated cyclin-Cdk1 complexes to phosphorylate T33-Sic1ΔC (B), T33H1-Sic1ΔC (C), or T33-Sic1ΔC-*1234rxl* (D). In the case of Clb cyclins, activity was also measured with hydrophobic patch-mutated cyclins (hpm, black bars). The dark gray bars in (B) and (C) denote the relative hp-dependent docking effect, and these values are bound to the right-hand scale of the Y axes. (E) The k_cat_/K_M_ profiles for the control substrate, the H1-based peptide PKTPKKAKKL. (F) The impact of different RXL motif-bearing docking sites on the phosphorylation specificity of T5, T33, and S76 was studied with Cln2-Cdk1 and Clb5-Cdk1, using substrate constructs with a single RXL motif left unmutated: Sic1ΔC, Sic1ΔC-*234rxl*, Sic1ΔC-*134rxl*, Sic1ΔC-*124rxl*, Sic1ΔC-*123rxl*, and Sic1ΔC-*1234rxl*.

We therefore suspected that the weak intrinsic activity of early cyclin-Cdk complexes is compensated by docking sites. However, a hydrophobic patch is not apparent in the primary sequence of Cln1, -2, or -3, and so it is not clear how Cln-Cdk1 complexes are able to efficiently phosphorylate their G1 targets. To address this question, we analyzed Cln-Cdk1 activity toward Sic1, a Cdk1 inhibitor and well-established target of Cln-Cdk1 at the G1/S transition. We constructed a version of Sic1 lacking the inhibitory C-terminal region (Sic1ΔC), and mutated the Cdk consensus motifs (S/TP) to alanines, except for the functionally essential site T33 (Nash et al., 2001). This construct allowed us to analyze the phosphorylation of a single site and quantitatively characterize individual specificity elements of the kinase. Studies of phosphorylation of the T33-Sic1ΔC protein and its derivatives revealed numerous important features of cyclin-Cdk1 specificity (Figures 2A–2E, Table S1). To allow direct comparison with specificity for the H1 control peptide motif, we also analyzed a control protein in which the H1 substrate sequence PKTPKKAKKL replaced the T33 substrate site (T33H1-Sic1ΔC).

First, we found that T33 in Sic1 is efficiently phosphorylated by Clb5-Cdk1 and that this specificity depends on the hydrophobic patch of the cyclin. Thus, T33-Sic1ΔC belongs to the Clb5-specific subset of Cdk1 targets that we previously described (Loog and Morgan, 2005). We also found that T33-Sic1ΔC was a relatively specific substrate for Cln2- and Clb3-Cdk1 when compared with the gradual H1 peptide phosphorylation profile (Figures 2A, 2B, and 2E). When the hydrophobic patch was mutated in the B-type cyclins (Figures 2B–2E, black bars) or when the four RXL motifs in Sic1ΔC (in positions 13–15, 89–91, 114–116, and 147–149) were mutated to alanines (T33-Sic1ΔC-*1234rxl*), the rate of phosphorylation by Clb5 and Clb3 decreased considerably, producing a rising specificity pattern for sequential Clb-Cdk1 complexes that is similar to that observed with the model peptide (Figure 2E). Remarkably, this rising specificity is accompanied by declining hydrophobic patch-dependent docking of B-type cyclins. This effect was calculated by dividing the k_cat_/K_M_ values for wild-type and hpm versions of the enzyme complexes (Figures 2B and 2C; dark gray bars). As we observed with peptide specificity, Clb3-Cdk1 showed an intermediate effect of the docking interaction as compared to Clb5- and Clb2-Cdk1. These data indicate that the early B-type cyclin-Cdk1 complexes can specifically phosphorylate Sic1 using the RXL-hp docking motif, while still being weak kinases for the optimal consensus site. Thus, a blend of increasing intrinsic activity and decreasing docking-site dependence results in roughly equal rates of T33-Sic1ΔC phosphorylation by all B-type cyclin-Cdk complexes, as expected given that the levels of Sic1 must be kept to a minimum from G1/S to mitotic exit. In contrast, for mitotic targets, whose specificity profile is exemplified by the model peptide (Figure 2E), the gradually increasing Cdk1 specificity peaks in mitosis, creating temporal separation of the phosphorylation relative to T33-Sic1ΔC-like targets.

In the case of Cln2-Cdk1, which does not seem to possess a conventional hydrophobic patch, mutation of the docking motifs in the Sic1 substrate caused about a 3- to 4-fold decrease in activity (compared with a 50-fold effect in the case of Clb5; Figures 2B and 2D, and Table S1).

To further understand the importance of the docking mechanism, we studied the impact of different potential RXL motifs on the phosphorylation of sites T5, T33, and S76 in Sic1. We used T5-Sic1ΔC, T33-Sic1ΔC, and S76-Sic1ΔC constructs with all the RXL motifs mutated to alanines, or with one motif left unmutated (Figure 2F). The results indicated that for Clb5 there are different designated RXL motifs for each phosphorylation site. RXL2 and to a lesser extent RXL3 enhance the phosphorylation of T5 and T33, while S76 is connected exclusively to RXL3. Cln2-Cdk1, in contrast, profits weakly but almost equally from each of the docking sites. This assumption is supported by the fact that a similar exclusive designation of the RXL docking site for Clb5-specific S phase targets has been shown previously for Fin1 (Loog and Morgan, 2005) and Orc6 (Archambault et al., 2005). The potential importance of the distance between the RXL docking site and the active site of the Cdk has been addressed previously in structural studies with cyclin A-Cdk2 (Cheng et al., 2006).

**Figure 3:**
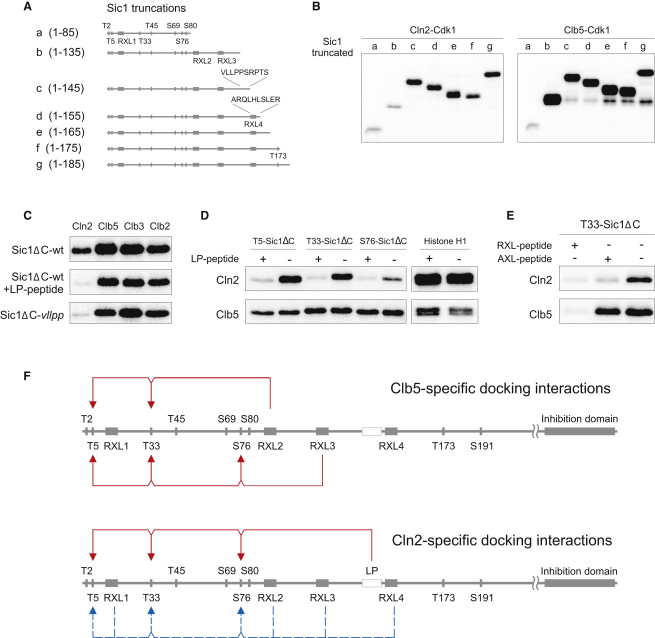
A Hydrophobic Stretch in Sic1 Enhances Cln2-Specific Phosphorylation Relative to Clb-Cdk1 (A and B) A 10 amino acid stretch that enhances the Cln2 specificity was located by systematic truncation of Sic1. (C) A five alanine mutation in the docking site of Sic1 (*vllpp* mutation) reduced the phosphorylation specificity for Cln2-Cdk1 but not for Clb5-Cdk1. Similar results were obtained using a competitor peptide based on the 10 amino acid stretch (the LP peptide, VLLPPSRPTS) between residues 136 and 145 as indicated in (A). (D) The LP peptide is equally effective in the inhibition of Cln2 specificity for sites T5, T33, and S76, as demonstrated with Sic1ΔC-based constructs described in Figure 2F. (E) The rate of Cln2-dependent phosphorylation of Sic1Δ can be reduced by addition of either RXL- or AXL-containing competitor peptides (based on the docking sequence of RVNRILFPT of Sic1: RVNRILFPT and RVNAIAFPT), whereas only the RXL peptide reduces the specificity of Clb5. (F) A scheme showing the observed docking interactions between Sic1 and Clb5 or Cln2. The dashed arrows indicate the mild cooperative effect of the leucines of the RXL motifs on Cln2 specificity.

Since the RXL motifs in T33-Sic1ΔC, when compared with the H1 peptide specificity profile, were only partly responsible for the relative Cln2 specificity, we searched for additional potential Cln2-specific docking sites by truncating the Sic1 molecule from its C terminus. We identified a 10 amino acid stretch that considerably enhanced the rate of Cln2-dependent phosphorylation, but not that of the Clbs (Figures 3A and 3B). The stretch contained a row of hydrophobic residues, and mutating the first five of them, VLLPP, to alanines (Sic1ΔC-*vllpp*) caused a considerable loss of the Cln2-dependent phosphorylation rate (Figure 3C). A synthetic peptide based on the 10 amino acid stretch was a competitive inhibitor of the docking, confirming that the effect of the deletion mutation was likely not due to a conformational anomaly in the mutant but a docking interaction between Cln2 and the substrate (Figure 3C). The specificity of the interaction was further confirmed by the observation that both the *vllpp* mutation and the peptide had an effect on Cln2, but not on Clb5. Cln1, which is closely related to Cln2, showed a similar LP peptide effect (data not shown). In contrast to the RXL docking sites used by Clb5, the LP interaction enhanced phosphorylation at all three sites in Sic1 almost equally (Figure 3D), suggesting that this is not a strictly distance-dependent docking but rather a general enhancement of the interaction between the substrate and the cyclin.

To locate the potential hydrophobic docking pocket in Cln2, we introduced *hpm*-like triple mutations into several candidate sites bearing some resemblance to the *hp* of B-type cyclins, as well as into the predicted *hp* itself. None of these mutations changed the specificity profile (data not shown), suggesting that there are several hydrophobic regions on the surface of Cln2 that contribute to the interaction. We also considered the possibility that the mild effect on Cln2 specificity that we observed with mutations in the RXL motifs (Figure 2F) was due to the removal of hydrophobic leucine residues. To test this, we compared the effects on Cln2 activity of a synthetic peptide based on the RXL2 motif of Sic1 (RVNRILFPT) with a similar peptide in which the arginine of the RXL motif was replaced with alanine (“AXL peptide”). Both peptides caused a 3- to 4-fold effect on Cln2-dependent phosphorylation of Sic1ΔC, whereas only the peptide with the intact RXL motif had an effect on Clb5-dependent phosphorylation (Figure 3E). A summary of docking interactions for Cln2-Cdk1 and Clb5-Cdk1 is presented in Figure 3F.

### Cyclins Modulate the Consensus Site Specificity Profile of Cdk1, Creating Distinct Optimal Profiles for Cln2- and Clb2-Cdk1

Next, we asked if, in addition to the observed docking interaction, some other mechanism might further enhance the specificity of Cln2. We noticed that many known physiological Cdk target sites, and also the H1 peptide, contain multiple P and K residues (e.g., PKTPKKAKKL). We wondered if these residues, while being important constituents of the Cdk1 consensus motif S/T-P-X-R/K, might also have a role in recognition and specificity when present in other nearby positions. To address the importance of proline and lysine residues in Cln2 specificity, we introduced these residues at different positions around T33 in the T33-Sic1ΔC construct. This system provides an advantage over traditional random peptide library techniques used for specificity studies of kinases (Mok et al., 2010), as it enables the study of docking effects and phosphorylation site primary structure requirements simultaneously in the context of a physiological protein target.

**Figure 4:**
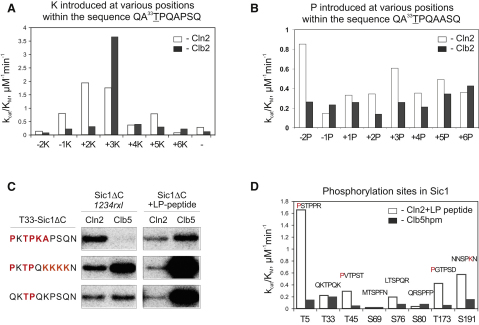
Cyclins Differentially Modulate the Phosphorylation Site Consensus Sequence of Cdk1 (A) Differential effect of lysines on the specificity profiles of Cln2-Cdk1 (open bars) or Clb2-Cdk1 (dark bars), using T33-Sic1ΔC variants with lysine introduced at various positions within the sequence QA^33^TPQAPSQ. (B) Differential effect of proline on the specificity profiles of Cln2-Cdk1 (open bars) or Clb2-Cdk1 (dark bars), using T33-Sic1ΔC variants with proline introduced at various positions within the sequence QA^33^TPQAASQ. (C) Examples of Cln2 versus Clb5 cyclin specificity, using different T33-Sic1ΔC and T33-Sic1ΔC-*1234rxl* variants bearing the motifs with highest differential specificity. The impact of LP motif-dependent specificity of Cln2 was determined with the LP competitor peptide. Red letters indicate the amino acid substitutions into different positions of the T33 site. (D) Cln2 versus Clb5 phosphorylation consensus site specificity profiles showing the docking-site-independent k_cat_/K_M_ values for eight different physiological phosphorylation sites of Sic1. Sic1ΔC constructs in which only the indicated single phosphorylation site is left unmutated were used in the phosphorylation assays. Cln2-Cdk1 combined with the LP competitor peptide and Clb5hpm-Cdk1 were used to eliminate the impact of the docking sites.

First, we analyzed the effects of adding lysine in different positions within the sequence motif QA^33^TPQAPSQ in the context of Sic1ΔC (Figure 4A), using Cln2-Cdk1 or Clb2-Cdk1. We found that Clb2 had a strong requirement for the lysine at position +3, the conventional position belonging to the consensus motif, while Cln2 exhibited specificity for lysine both at position +2 and +3. Importantly, the Lys+2 was exclusively specific for Cln2 over Clb2 and the two other B-type cyclins (Table S1). Variation of proline in the same manner, within a template sequence motif QA^33^TPQAASQ, revealed a positive element for Cln2 and Clb2: a proline at position −2 (Figure 4B).

Data from comparative analyses (Table S1) suggest that in the case of B-type cyclins lacking the hydrophobic patch, the overall substrate phosphorylation efficiency toward the T-P motif changes gradually as described above for the model peptide, and exhibits little variation among Clb5, Clb3, and Clb2. Clb specificity relative to Cln2 can be attained by simultaneously introducing multiple basic residues on the right-hand side of the T-P motif as well as a proline in position −2. Substrates containing a combination of these specificity elements follow the pattern of gradually rising mitotic specificity described in Figure 1, with the specificity for Cln2-Cdk1 being the lowest and for Clb2-Cdk1 the highest (e.g., H1 motif, PKTPQKKKK, PKTPKK; Table S1). On the other hand, the specificity of Cln2-Cdk1 relative to B-type cyclins can be enhanced by introducing lysine at position +2 and avoiding it at position +3. Figure 4C provides examples of the sharp differences that can be obtained in Cln2 versus Clb5 specificity. The *1234rxl* mutation or the LP competitor peptide was used to assess the docking site-independent fraction of the specificity of Cln2- or Clb5-Cdk1. This abrupt change of specificity may be used in switches after the G1/S transition, when Cln2 is degraded and the system needs to be adjusted from G1 to S phase mode.

To demonstrate that the specificity profiles we determined above are also reflected in phosphorylation of physiologically important Cdk1 sites, we determined the differential specificity of Cln2-Cdk1 and Clb5-Cdk1 for eight sites of Sic1 (Figure 4D). Indeed, the four most Cln2-specific sites (T5, T45, T173, and S191) contained Cln2-specific motifs.

### Cyclin Specificity Determinants Are Important In Vivo

To test the validity of the gradual cyclin specificity model in vivo, we set up a system to follow the cyclin-specific phosphorylation of nondestructible versions of Sic1ΔC in cells released from a G1 arrest. Cells expressing one of two different Sic1 mutants were studied. Either the consensus motif P-X-T-P-X-K, specific for both Cln2 and Clbs, or the Cln2-specific motif P-X-T-P-K-A was introduced at all four of the Cdk sites T5, T33, T45, and S76. The rest of the Cdk1 sites were mutated to alanines, and site T2 was left unchanged. We examined the dynamics of phosphorylation of these proteins in vivo by measuring phosphorylation-dependent mobility shifts on Phos-Tag SDS-PAGE gels (Figure S1A). We also confirmed that the two constructs had the predicted cyclin specificity in vitro (Figure S1D). Using a Sic1ΔC construct in which all Cdk sites were mutated (Sic1ΔC-9A), and also by specific chemical inhibition of Cdk1, we confirmed that increasing phosphorylation of Sic1 as cells entered the cell cycle was caused by Cdk1 activity (Figures S1A–S1C). The non-Cdk1-dependent fraction of the shifts did not change over the cell cycle (Figure S1C), and the kinase(s) responsible for these shifts remains to be identified. Several kinases other than Cdk1 (Pho85, Hog1, and CK2) may phosphorylate Sic1 (Coccetti et al., 2006; Escote et al., 2004; Nishizawa et al., 1998).

**Figure 5:**
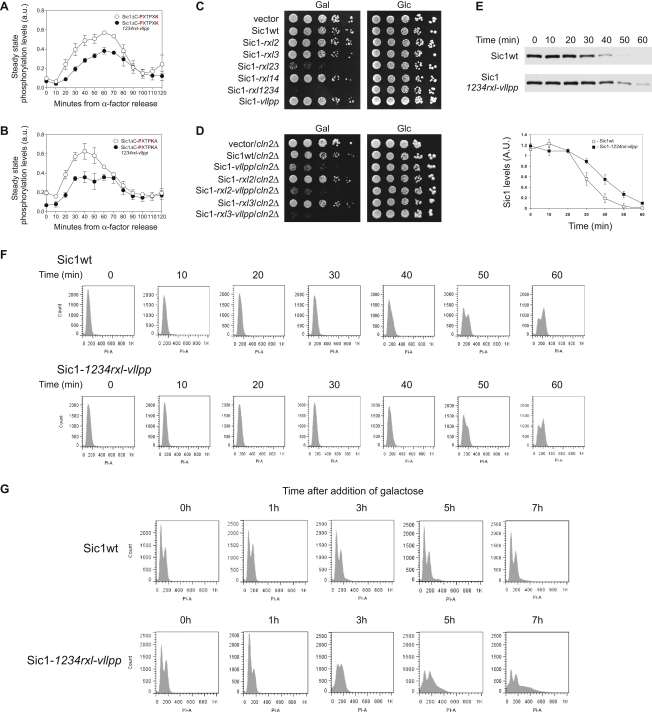
Analysis of Changes in Cdk1 Substrate Specificity during the Cell Cycle and of the Physiological Importance In Vivo of Different Substrate Docking Sites (A and B) Cells constitutively expressing the indicated Sic1ΔC-3HA construct were released from α factor arrest, and at the indicated time points Phos-Tag SDS-PAGE and western blotting were used to assess phosphorylation state (see Figure S1). The constructs contained either the Clb2-specific P-X-T-P-X-K motif (A) or the Cln2-specific P-X-T-P-K-X motif (B) at positions T5, T33, T45, and T76. Other Cdk1 sites were mutated to alanines, and T2 was left unchanged. As controls, we also tested the same constructs with mutations of the LP and the RXL docking sites. Sic1 bands on the western blot were scanned using the GelDoc (GE), and each phosphorylated species was quantified. The relative steady-state phosphorylation at each time point was calculated as a ratio of pS/S_T_, where S_T_ is the total signal of Sic1ΔC-3HA and pS is the total signal of phosphorylated bands multiplied by the number of phosphates each band contains. See also Figure S1. Analyses were performed in duplicates, and error bars indicate standard error of the mean. (C and D) The importance of Clb5-specific RXL motifs (C) and the Cln2-specific LP motif (D) in the degradation of Sic1 was tested by overexpressing different Sic1 constructs under the *GAL1* promoter. To assess the effect of the LP docking site, the strain used in (D) was sensitized by reducing Cln-Cdk1 activity in the cell by deleting Cln2. (E) Western blotting analysis of Sic1 levels after the release of cells from a G1 arrest. Strains carrying CEN vectors with *GAL*-*SIC1* or *GAL*-*SIC1*-*1234rxl* -*vllpp* were arrested in G1 with α factor for 2.5 hr. *SIC1* expression was induced in the arrested cells by addition of galactose for 45 min. Both galactose and α factor were removed and the cells were released into glucose-containing medium. Sic1-3HA levels were analyzed at different time points using western blotting. In the lower panel the combined quantified Sic1 profiles from two independent experiments are presented. The error bars indicate standard error of the mean. (F) To demonstrate how altered Cdk1 specificity toward Sic1 affects the timing of DNA replication, we performed flow cytometry of DNA content for cells taken at different time points of the α factor release experiment presented in (E). (G) To explore further how altered Cdk1 specificity toward Sic1 affects the control of DNA replication, we performed flow cytometry of DNA content in asynchronous cells expressing the Sic1 mutant lacking cyclin docking motifs. Strains carrying CEN vectors with *GAL-SIC1* or *GAL-SIC1-1234rxl-vllpp* were grown to log phase, and the expression of Sic1 was initiated by addition of galactose. Cells expressing Sic1 with mutations in the docking sites caused DNA rereplication (DNA > 2N). Cells expressing wild-type Sic1 did not exhibit any signs of rereplication.

Profiles of total Sic1 phosphorylation in vivo, plotted in Figures 5A and 5B, revealed that the phosphorylation status of the construct bearing the P-X-T-P-X-K motifs reached half-maximal levels at early time points, compared to the construct with the same consensus motifs but containing the *1234rxl/vllpp* mutations in the docking sites. The latter construct showed a delayed accumulation of phosphorylated forms with a half-maximum at 40–50 min, corresponding roughly to the onset of mitosis. This result is in agreement with the kinetic data presented earlier. The docking sites, however, compensated for the low activity in the early cycle. As shown in our earlier results in vitro, the relative effect of the docking sites decreased at the late time points, indicating that Clb2 gains relatively less from the docking sites than the earlier complexes. However, since the cyclin peaks exhibit considerable overlap (Figure 1A), the changing effects of the docking sites in the time course experiments are not as sharp as the differences in kinetic constants of individual cyclin-Cdk1 complexes.

The construct bearing the Cln2-specific motif P-X-T-P-K-A showed a different profile in which an early maximal activity was followed by a slight decline as the cells progressed toward mitosis. Analogously to the previous case, the *1234rxl/vllpp* mutations lowered the phosphorylation equilibrium, and the relative effect of these mutations decreased with time. These experiments outline the physiological relevance of the major elements of the gradual cyclin specificity model in vivo.

Further demonstrating the physiological importance of substrate docking sites, we found that overexpression of full-length Sic1-*23rxl* or Sic1-*1234rxl* severely reduced the viability of yeast cells, while the overexpression of Sic1-wt had little effect (Figure 5C). In contrast, the *vllpp* mutation in Sic1 did not affect the growth of cells. However, Cln2-Cdk1 and Clb5-Cdk1 still seem to cooperate in the phosphorylation of Sic1, as the *vllpp* mutation was found to affect the growth of *cln2Δ* cells (Figure 5D), probably because the reduced Cdk1 activity in these cells makes them more sensitive to decreased Cln-dependent Sic1 phosphorylation.

To further confirm that the change in Sic1 phosphorylation specificity is accompanied by a change of Sic1 function in vivo, we demonstrated that expression of a version of Sic1 with mutated cyclin docking sites resulted in delayed Sic1 degradation and entry into S phase (Figures 5E and 5F). Furthermore, overexpression of a Sic1 mutant with mutated docking sites caused DNA rereplication (Figure 5G), suggesting that the docking-site-enhanced Cdk1 substrate specificity toward the Cdk1 inhibitor Sic1 has been, at least partially, evolved to ensure the irreversibility of G1/S transition by promoting potent and continuous suppression of Sic1. We propose that this irreversibility is important throughout S phase, as any erroneous bursts of late Sic1 expression would inhibit Clb-Cdk1 complexes and allow origin relicensing and rereplication.

### A Screen for Cyclin-Specific Cdk1 Targets Reveals Proteins with Different Specificity for Each of Four Representative Cyclin-Cdk1 Complexes

**Figure 6:**
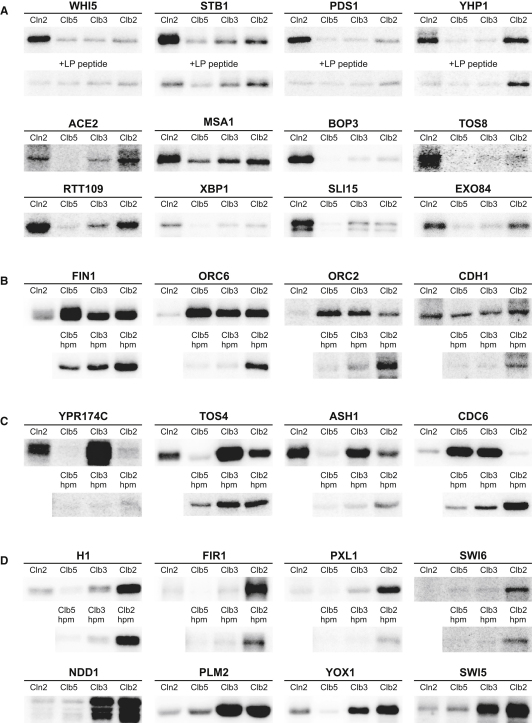
Phosphorylation Screen of Cyclin-Specific Cdk1 Targets Purified protein substrates were tested in a standard kinase assay using four representative complexes of cyclin-Cdk1. The apparent k_cat_/K_M_ values are provided in Table S2. (A) Type I substrates specific for Cln2-Cdk1. Lower panels indicate activity in the presence of the competitor peptide based on the LP docking site of Sic1. (B) Type II substrates specific for Clb5- and Clb3-Cdk1. Lower panels indicate activity with the hpm versions of each Clb. (C) Type III substrates showing hp-dependent specificity for Clb3 and no specificity for Clb5 and/or Clb2. (D) Gradual specificity profile of the “mitotic targets” (Type IV substrates) presented together with selected examples of Clb-hpm-Cdk1 profiles. See also Table S2.

Next, we explored how cyclin-dependent changes in Cdk1 specificity are reflected in other physiological targets of Cdk1. We developed methods for a quantitative Cdk1 substrate screen, with a special emphasis on searching for Cln2- and Clb2-specific physiological targets. Interestingly, when examining the reported targets of Cln-Cdk1, we noted that they are all multiphosphorylated proteins. We therefore analyzed Cln2-Cdk1 activity with several candidate multisite Cdk1 targets that were chosen with an emphasis on their potential functional connection to Cln2 (Horak et al., 2002; Sundin et al., 2004), and on the condition that they contain at least five Cdk sites. We included a number of known Cln2 and Clb targets as well as several uncharacterized ORFs with at least five Cdk sites. Potential targets were expressed and purified from bacterial expression systems, and those with reasonable yields were submitted to specificity analyses with the four cyclin-Cdk1 complexes. Relative k_cat_/K_M_ values obtained from the phosphorylation experiments (Table S2), with examples presented in Figure 6, revealed several types of cyclin specificity profile. We grouped targets into four types.

Type I substrates are proteins with high Cln2 specificity or Cln2 and Clb(3)2 specificity (Figure 6A). Among these targets was Whi5, the repressor of the G1-specific SBF transcription factor and the analog of mammalian pRB in budding yeast. The screen revealed a number of other Cln2-specific targets involved in G1-specific transcriptional control, including Xbp1, Xhp1, and Tos8 (Figure 6A and Table S2). Strikingly, we demonstrated that the Cln2 specificity for several targets was largely dependent on the LP docking site, as the presence of the LP competitor peptide reduced the phosphorylation signal for Cln2 but not for Clbs. These results suggest that the LP docking interaction may have broad significance and can be used by many Cln-Cdk1 targets. The amino acid sequences of these proteins contain several hydrophobic regions whose sequence is reminiscent of the LP site in Sic1.

The second substrate type was defined as Clb5-specific targets whose specificity depends on hydrophobic patch docking as described by us previously (Loog and Morgan, 2005). These proteins have specificity for Clb5, but they may also be specific for Clb3, exhibiting an intermediate level of hp dependency as described above in Figure 2A for T33-Sic1ΔC. This group includes the spindle-stabilizing protein Fin1, which must be fully phosphorylated early in the cell cycle to prevent it from binding to the spindle (Woodbury and Morgan, 2007). In addition, we found one target (the Cdk1 inhibitor Far1, Table S2) that showed specificity for Cln2 and Clb5, but not for Clb3 and Clb2.

The third type of Cdk target was defined as those showing hp-dependent specificity for Clb3 and no specificity for Clb5 and/or Clb2 (Figure 6C). This was a surprising finding, as there are no previous reports of Clb3-Cdk1-specific substrates or functions. This group included the putative transcription factor Tos4, the transcription factor Ash1, and a protein of unknown function, YPR174C. Among these targets was the replication factor and pre-RC component Cdc6, which shared the characteristics of type II and III substrates and showed a sharp specificity difference between Clb3 and Clb2. We found that this specificity was due to the efficient inhibitory interaction of Cdc6 with Clb2-Cdk1, but not with Clb5- and Clb3-Cdk1 (our unpublished data).

The fourth type of Cdk target was defined as “Clb2-specific” or mitotic substrates. These proteins followed the characteristic gradual cyclin specificity pattern outlined in the model peptide studies above. One example of this type was Ndd1, a component of the transcription factor complex controlling the expression of G2/M-specific genes, including *CLB2* itself (Darieva et al., 2006). Ndd1 is known to be phosphorylated and thereby activated by the polo kinase Cdc5, which uses phosphorylated Cdk sites as docking elements (Asano et al., 2005; Snead et al., 2007). We predict that Clb2-specific phosphorylation might accelerate the Cdc5-dependent secondary phosphorylation, thus creating positive feedback in G2-specific gene transcription. Additionally, the important cell cycle-related transcription factor Swi6 belongs to the type IV category.

Since the screen was performed on multisite Cdk targets, we wondered if the specificity profiles might reflect the consensus site motifs of the phosphorylation sites in these proteins. We found that the targets showing the strongest gradual mitotic pattern (hp-independent) (Ndd1, Cdc6, Orc2, Plm2, Swi6, and Fir1) all contained one to two sites with the H1-like mitotic consensus sequence P-X-S/T-P-X-[K/R]_n > 1_, which contains more than one K/R residue within the six C-terminal positions from the phosphorylation site. On the other hand, in the Cln2-specific targets, the positive determinants +2K/R, −2P, and the prolines in other C-terminal positions were found to be frequent. For example, one of the most potent Cln2-specific targets, Stb1, contains four strong Cln2-specific sites bearing the motifs P-X-S/T-P-K/R-X, P-X-S/T-P-X-X, P-X-S/T-P-P-X, or X-X-S/T-P-K/R-X, but no sites with the mitotic motif P-X-S/T-P-[K/R]_n > 1_. It is important to remember, however, that it is difficult to extrapolate our studies of consensus motifs to multisite substrates, because different phosphorylation sites may not be similarly accessible. In such cases, a few accessible sites may largely determine the net specificity despite the presence of other motifs in the primary sequence of the protein.

## Discussion

**Figure 7:**
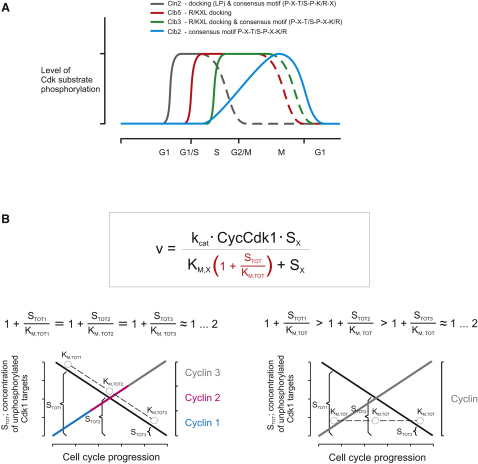
Dynamics of Cdk1 Substrate Specificity during the Cell Cycle (A) A schematic view of the dynamic changes of Cdk1 specificity during the cell cycle. (B) Modified Michaelis-Menten formula with the highlighted inhibitory term describing how the Cdk1 substrate pool (S_TOT_) can act as a competitive inhibitor relative to any particular substrate (S_X_). The lower panel on the left side presents a schematic view of a hypothetical system based on sequential accumulation of three cyclins with gradually changing specificity, and the panel on the right side presents a similar system with a single mitotic cyclin (rising linear lines). The gradual phosphorylation and decrease of the unphosphorylated Cdk1 substrate pool during the progression of the cell cycle is depicted as the declining black line. In the three-cyclin system, the inhibitory terms (1 + S_TOT_/K_M,TOT_) are kept relatively low and equal throughout the course of the cyclin synthesis, because as the substrate pool gets gradually phosphorylated S_TOT_ for each cyclin decreases in correlation with K_M,TOT_. In the system with a single cyclin, the inhibitory term is high in the early stages of the cell cycle, potentially delaying timely phosphorylation of premitotic targets.

In this study, we sought a quantitative understanding of dynamic changes in Cdk1 specificity over the budding yeast cell cycle. We found that a gradual change of specificity is an intrinsic feature of the cyclin-Cdk1 system, and it seems to have evolved to prevent Cdk1 from prematurely triggering mitosis using the built-in delay mechanisms created by the weaker activity of the earlier cyclin-Cdk1 forms toward Clb2-specific mitotic targets. This weak activity, however, does not prevent the phosphorylation of G1 and S phase Cdk substrates, whose targeting is accomplished by RXL-hydrophobic patch interactions (Clb5, Clb3), by a hydrophobic LP docking site (Cln2), or by different consensus sites (Cln2) (Figure 7A). Thus, as a general conclusion, we can state that Cdk1 specificity is periodically changing in the course of the cell cycle.

Throughout the eukaryotes, mitotic Cdk1 activity is regulated in part by inhibitory phosphorylation by the tyrosine kinase Wee1. The homolog of Wee1 in budding yeast, Swe1, has been shown to exhibit a differential ability to phosphorylate and inactivate different cyclin-Cdk1 complexes, with the highest inhibitory potency toward Clb2 and gradually lower for the earlier cyclin-Cdks (Hu and Aparicio, 2005; Keaton et al., 2007). We determined that the relative differences in Swe1 specificity are about the same order of magnitude as the gradual change in the intrinsic specificities of Cdk1 (Figure S2). These data reveal another remarkable gradual phenomenon in the cyclin-Cdk1 system in yeast. The identity of the cyclins in complex with Cdk1 is sensed by the Swe1 kinase, which then applies inhibitory pressure in proportion to the intrinsic specificity of the complex. In fact, the same structural elements may control the accessibility of the active site of different cyclin-Cdk1s for substrate and for the kinase domain of Swe1.

The cyclin specificity model is an alternative to the quantitative model of the cyclin response, according to which different levels of accumulating Cdk1 activity trigger different cell cycle events (Coudreuse and Nurse, 2010; Stern and Nurse, 1996). The major weakness of a system behaving solely according to the quantitative model is that the temporal resolution of events depends entirely on the use of substrates with wide differences in specificities toward cyclin-Cdk1. According to this model, early events of the cell cycle would be switched on by optimal substrates that are extensively phosphorylated at low Cdk1 activity levels, while later events must be triggered by suboptimal substrates that are phosphorylated only at high kinase levels. For this model to work, the differences between the cyclin levels triggering S phase and M phase must be very large, with S phase triggered by a small fraction of the mitotic Cdk1 activity levels. This would apparently make S phase very vulnerable to even mild deviations and fluctuations of the cyclin signal, which could lead to premature initiation of later events.

While the quantitative model is apparently not sufficient to describe the function of cyclins, it also appears that the other extreme, according to which docking mechanisms are used throughout the cycle, is not correct either. Instead, each cyclin-Cdk1 complex in the sequence has improved intrinsic specificity culminating with the mitotic complex, which relies almost entirely on the intrinsic consensus-site specificity and minimally on docking sites. Thus, Cdk1 broadens its specificity gradually for wider and wider fractions of the proteome.

With several hundred Cdk1 targets in the cell, most of which contain multiple Cdk1 consensus sites, the total substrate concentration for Cdk1 could be hundreds of micromolar or even low millimolar. This target pool is unphosphorylated in early G1 phase, and because multiple targets of an enzyme act as competitive inhibitors relative to one another, the higher K_M_ values prevent early cyclin-Cdk1 complexes from being inhibited by the pool of unphosphorylated targets, as illustrated in Figure 7B. The highlighted inhibition term (1 + S_TOT_/K_M,TOT_) in the modified Michaelis-Menten equation raises the apparent K_M_ for any given substrate and thereby decreases its phosphorylation rate. The panel on the left side of Figure 7B schematically describes a simplified system following the cyclin specificity model, with three cyclins synthesized in sequence. As the concentration of the bulk unphosphorylated Cdk1 substrate pool decreases in correlation with the K_M,TOT_ values (phosphorylated residues have considerably lower affinity for the active site), the inhibition term is kept at a constant low level, allowing each complex to perform its specific function using the cyclin-specific docking sites, while being unhindered by the bulk substrate pool, at the point of the cycle where it is assigned. The panel on the right side of Figure 7B shows the system behaving according to the quantitative model, based on accumulation of a single cyclin similar to mitotic cyclin 3 with respect to specificity. In such a system, a full phosphorylation rate of any target is achieved only after substantial accumulation of cyclin when a large part of the total Cdk substrate pool is phosphorylated and the inhibition term is minimal.

We speculate that cyclin specificity evolved as follows. Early eukaryotic cell cycle control depended on a single cyclin system, in which S phase was switched on by low levels of kinase activity, using substrates bearing the optimal consensus motifs (S/TPXK/R), after which higher kinase levels triggered M phase by phosphorylation of suboptimal motifs (S/TP). Due to a relatively low complexity of regulation, there were few substrates and little substrate competition. Eventually, evolution of cyclin docking sites (LP and/or RXL) provided additional affinity for S phase targets carrying cyclin docking motifs in addition to the optimal consensus phosphorylation site. At this stage, more regulatory complexity also evolved, requiring more substrate sites and therefore more competition and mutual inhibition by substrates in S phase. This competition limited the possibilities for further complexity, until multiple cyclins appeared with weaker earlier complexes to reduce the competition at the active site level. As earlier cyclins became weaker activators of Cdk1 (higher K_M_), early targets with docking motifs, like Sld2 and Sic1, would be more rapidly phosphorylated as competition from optimal substrates decreased. Thus, as a result of cyclin docking interactions, suboptimal intrinsic specificity became an advantage, allowing greater complexity in Cdk-triggered processes and control systems. Such a system can survive (with some difficulty) using a single mitotic cyclin, which will hit the S phase targets (containing S/TPXK/R motifs) earlier than the later targets (containing S/TP motifs), because the former ones have lower K_M_s and are able to outcompete the latter ones.

The most surprising outcome of our work is that both Clb- and Cln2-Cdk1 possess some strikingly different elements in their phosphorylation site consensus sequence. While the general activity of a protein kinase has been shown to be regulated in a wide variety of cases, the modulation of the primary structure specificity profile by a regulatory subunit has to our knowledge not been reported. Additionally, Cln2 specificity was found to be strongly enhanced by a previously unreported docking interaction involving a hydrophobic stretch in Sic1. While the precise structural motifs of this docking interaction are yet to be established, we speculate that a hydrophobic pocket on the cyclin could serve as a docking site for this motif.

The concept of dynamically changing Cdk1 specificity could be used to explain previously reported phenotypes obtained by genetic manipulations of cyclin genes in yeast. For example, it may explain why yeast cells lacking both Clb5 and Clb6 experience only an S phase delay (Schwob and Nasmyth, 1993), while double mutants of Clb2 and Clb3 or Clb2 and Clb1 are inviable and arrest prior to mitosis (Mendenhall and Hodge, 1998). In the first case, after a delay, the intermediate Clb3-Cdk1 and the intrinsically most potent Clb2-Cdk1 can phosphorylate the S phase targets at a reasonable rate. In the two last-mentioned cases, however, the poor ability of early B-type cyclins to promote the phosphorylation of Clb2-specific targets apparently makes the cells incapable of initiating mitosis. Perhaps for the same reason, partly stabilized Clb5 is unable to block mitotic exit (Wasch and Cross, 2002), when compared with a strain overexpressing stabilized Clb2 (Surana et al., 1993). Interestingly, our cyclin specificity model also fits well with the observations that fully stabilized Clb5 is still capable of mitotic exit but incapable of S phase initiation, which we propose is due to constant Clb5-specific phosphorylation of S phase targets of the preRC, preventing origin licensing (Sullivan et al., 2008). We also propose that blocking Clb2 activity toward Clb5 targets may be important at certain stages of mitosis (e.g., for the dephosphorylation switch of “Fin1-like” targets [Woodbury and Morgan, 2007]) and could be accomplished, again, by the inability of Clb2 to use the RXL-hp docking mechanism.

It will be important to determine if higher eukaryotes possess a similar dynamic specificity scheme. The general conservation of the gradual model remains to be shown, but our studies of budding yeast shed light on somewhat puzzling and unexpected cyclin knockout studies in mice: if a cyclin is deleted, the process it was meant to trigger is delayed until the activated Cdk, through the synthesis of other accumulating cyclins, reaches the levels where the net value of (k_cat_/K_M_)^∗^[E] corresponds to the threshold of the trigger. If one deletes the weaker early cyclins, compensation by the later, stronger ones is more likely than the opposite situation, as mice lacking cyclins E and D have been shown to be viable, while cyclins B1 and A2, for example, are the most nonredundant of all cyclins and are required for embryo viability (Satyanarayana and Kaldis, 2009).

In conclusion, we have shown that in the course of the cell cycle, different cyclins gradually change the substrate specificity of Cdk1 at the active-site level. This modulation of specificity, when combined with docking site interactions, reveals the dynamic nature of continuous specificity changes of Cdk1 in the course of the cell cycle and provides a wide range of selective switchpoints for different cell cycle transitions.

## Experimental Procedures

The TAP method was applied for purification of cyclin-Cdk1 complexes and Swe1 as described previously for Clb5-TAP-Cdk1 and Clb2-TAP-Cdk1 (Puig et al., 2001; Ubersax et al., 2003). 3HA-Cln2-Cdk1 was purified according to published protocols (McCusker et al., 2007). 6His-tagged recombinant T33-Sic1ΔC constructs and substrates were purified by cobalt affinity chromatography. GST-tagged substrates were purified on glutathione agarose columns.

For the quantitative phosphorylation assays of T33-Sic1ΔC constructs and recombinant substrates, substrate concentrations were kept in the range of 0.5–2 μM (in the linear [S] versus v_0_ range, several-fold below the estimated K_M_ value), and initial velocity conditions were defined as an initial substrate turnover of up to 10% of the total turnover. For the steady-state peptide kinetics of the Histone peptide PKTPKKAKKL, a similar assay composition was used as for protein substrates, and phosphocellulose paper was used for the quantification of the phosphorylated substrate.

For the western blotting experiments using the Phos-Tag SDS-PAGE, the Sic1ΔC-3HA versions were cloned into vector pRS315 and constitutively expressed under the *ADH* promoter. The cells were treated for 2.5 hr with 1 μg/ml α factor and released by washing. After the 50 min time point, α factor was readded to collect the cells in the next G1. The cells were lysed by bead beating in lysis buffer containing urea. Blotting of Phos-Tag SDS-PAGE gels was performed using a dry system iBlot (Invitrogen).

For viability assays on galactose plates and for galactose-induced expression time courses, Sic1 mutants were cloned into vector pRS416 under the *GAL1* promoter. For flow cytometry experiments, the DNA was stained with propidium iodide and the analysis was performed on a Becton Dickinson BD LSRII flow cytometer.

## Supplemental Information

Document S1. Two Figures, Four Tables, Supplemental Discussion, Supplemental Experimental Procedures, and Supplemental References

## References

[bib1] Archambault V., Buchler N.E., Wilmes G.M., Jacobson M.D., Cross F.R. (2005). Two-faced cyclins with eyes on the targets. Cell Cycle.

[bib2] Asano S., Park J.E., Sakchaisri K., Yu L.R., Song S., Supavilai P., Veenstra T.D., Lee K.S. (2005). Concerted mechanism of Swe1/Wee1 regulation by multiple kinases in budding yeast. EMBO J..

[bib3] Booher R.N., Deshaies R.J., Kirschner M.W. (1993). Properties of Saccharomyces cerevisiae wee1 and its differential regulation of p34CDC28 in response to G1 and G2 cyclins. EMBO J..

[bib4] Cheng K.Y., Noble M.E., Skamnaki V., Brown N.R., Lowe E.D., Kontogiannis L., Shen K., Cole P.A., Siligardi G., Johnson L.N. (2006). The role of the phospho-CDK2/cyclin A recruitment site in substrate recognition. J. Biol. Chem..

[bib5] Coccetti P., Zinzalla V., Tedeschi G., Russo G.L., Fantinato S., Marin O., Pinna L.A., Vanoni M., Alberghina L. (2006). Sic1 is phosphorylated by CK2 on Ser201 in budding yeast cells. Biochem. Biophys. Res. Commun..

[bib6] Coudreuse D., Nurse P. (2010). Driving the cell cycle with a minimal CDK control network. Nature.

[bib7] Cross F.R., Jacobson M.D. (2000). Conservation and function of a potential substrate-binding domain in the yeast Clb5 B-type cyclin. Mol. Cell. Biol..

[bib8] Cross F.R., Yuste-Rojas M., Gray S., Jacobson M.D. (1999). Specialization and targeting of B-type cyclins. Mol. Cell.

[bib9] Cross F.R., Archambault V., Miller M., Klovstad M. (2002). Testing a mathematical model of the yeast cell cycle. Mol. Biol. Cell.

[bib10] Darieva Z., Bulmer R., Pic-Taylor A., Doris K.S., Geymonat M., Sedgwick S.G., Morgan B.A., Sharrocks A.D. (2006). Polo kinase controls cell-cycle-dependent transcription by targeting a coactivator protein. Nature.

[bib11] Donaldson A.D. (2000). The yeast mitotic cyclin Clb2 cannot substitute for S phase cyclins in replication origin firing. EMBO Rep..

[bib12] Escote X., Zapater M., Clotet J., Posas F. (2004). Hog1 mediates cell-cycle arrest in G1 phase by the dual targeting of Sic1. Nat. Cell Biol..

[bib13] Fisher D.L., Nurse P. (1996). A single fission yeast mitotic cyclin B p34cdc2 kinase promotes both S-phase and mitosis in the absence of G1 cyclins. EMBO J..

[bib14] Horak C.E., Luscombe N.M., Qian J., Bertone P., Piccirrillo S., Gerstein M., Snyder M. (2002). Complex transcriptional circuitry at the G1/S transition in Saccharomyces cerevisiae. Genes Dev..

[bib15] Hu F., Aparicio O.M. (2005). Swe1 regulation and transcriptional control restrict the activity of mitotic cyclins toward replication proteins in Saccharomyces cerevisiae. Proc. Natl. Acad. Sci. USA.

[bib16] Ikui A.E., Archambault V., Drapkin B.J., Campbell V., Cross F.R. (2007). Cyclin and cyclin-dependent kinase substrate requirements for preventing rereplication reveal the need for concomitant activation and inhibition. Genetics.

[bib17] Keaton M.A., Bardes E.S., Marquitz A.R., Freel C.D., Zyla T.R., Rudolph J., Lew D.J. (2007). Differential susceptibility of yeast S and M phase CDK complexes to inhibitory tyrosine phosphorylation. Curr. Biol..

[bib18] Loog M., Morgan D.O. (2005). Cyclin specificity in the phosphorylation of cyclin-dependent kinase substrates. Nature.

[bib19] McCusker D., Denison C., Anderson S., Egelhofer T.A., Yates J.R., Gygi S.P., Kellogg D.R. (2007). Cdk1 coordinates cell-surface growth with the cell cycle. Nat. Cell Biol..

[bib20] Mendenhall M.D., Hodge A.E. (1998). Regulation of Cdc28 cyclin-dependent protein kinase activity during the cell cycle of the yeast Saccharomyces cerevisiae. Microbiol. Mol. Biol. Rev..

[bib21] Mok J., Kim P.M., Lam H.Y., Piccirillo S., Zhou X., Jeschke G.R., Sheridan D.L., Parker S.A., Desai V., Jwa M. (2010). Deciphering protein kinase specificity through large-scale analysis of yeast phosphorylation site motifs. Sci. Signal..

[bib22] Morgan D.O. (2007). The Cell Cycle: Principles of Control.

[bib23] Nash P., Tang X., Orlicky S., Chen Q., Gertler F.B., Mendenhall M.D., Sicheri F., Pawson T., Tyers M. (2001). Multisite phosphorylation of a CDK inhibitor sets a threshold for the onset of DNA replication. Nature.

[bib24] Nguyen V.Q., Co C., Li J.J. (2001). Cyclin-dependent kinases prevent DNA re-replication through multiple mechanisms. Nature.

[bib25] Nishizawa M., Kawasumi M., Fujino M., Toh-e A. (1998). Phosphorylation of sic1, a cyclin-dependent kinase (Cdk) inhibitor, by Cdk including Pho85 kinase is required for its prompt degradation. Mol. Biol. Cell.

[bib26] Puig O., Caspary F., Rigaut G., Rutz B., Bouveret E., Bragado-Nilsson E., Wilm M., Seraphin B. (2001). The tandem affinity purification (TAP) method: a general procedure of protein complex purification. Methods.

[bib27] Satyanarayana A., Kaldis P. (2009). Mammalian cell-cycle regulation: several Cdks, numerous cyclins and diverse compensatory mechanisms. Oncogene.

[bib28] Schwob E., Nasmyth K. (1993). CLB5 and CLB6, a new pair of B cyclins involved in DNA replication in Saccharomyces cerevisiae. Genes Dev..

[bib29] Snead J.L., Sullivan M., Lowery D.M., Cohen M.S., Zhang C., Randle D.H., Taunton J., Yaffe M.B., Morgan D.O., Shokat K.M. (2007). A coupled chemical-genetic and bioinformatic approach to Polo-like kinase pathway exploration. Chem. Biol..

[bib30] Stern B., Nurse P. (1996). A quantitative model for the cdc2 control of S phase and mitosis in fission yeast. Trends Genet..

[bib31] Sullivan M., Holt L., Morgan D.O. (2008). Cyclin-specific control of ribosomal DNA segregation. Mol. Cell. Biol..

[bib32] Sundin B.A., Chiu C.H., Riffle M., Davis T.N., Muller E.G. (2004). Localization of proteins that are coordinately expressed with Cln2 during the cell cycle. Yeast.

[bib33] Surana U., Amon A., Dowzer C., McGrew J., Byers B., Nasmyth K. (1993). Destruction of the CDC28/CLB mitotic kinase is not required for the metaphase to anaphase transition in budding yeast. EMBO J..

[bib34] Ubersax J.A., Woodbury E.L., Quang P.N., Paraz M., Blethrow J.D., Shah K., Shokat K.M., Morgan D.O. (2003). Targets of the cyclin-dependent kinase Cdk1. Nature.

[bib35] Wasch R., Cross F.R. (2002). APC-dependent proteolysis of the mitotic cyclin Clb2 is essential for mitotic exit. Nature.

[bib36] Woodbury E.L., Morgan D.O. (2007). Cdk and APC activities limit the spindle-stabilizing function of Fin1 to anaphase. Nat. Cell Biol..

