# Supplementary material for: Dynamics of Cdk1 Substrate Specificity during the Cell Cycle
Source: Mol Cell. 2011 Jun 10;42(5-4):610–23. doi: 10.1016/j.molcel.2011.05.016 (PMC3115021; doi:10.1016/j.molcel.2011.05.016)
Supplement: Document S1. Two Figures, Four Tables, Supplemental Discussion, Supplemental Experimental Procedures, and Supplemental References [file mmc1.pdf]

Molecular Cell, Volume *42*

Supplemental Information

Dynamics of Cdk1 Substrate Specificity during the Cell Cycle

Mardo Kõivomägi, Ervin Valk, Rainis Venta, Anna Iofik, Martin Lepiku, David O. Morgan, and Mart Loog

Inventory of Supplemental Information

Figure S1. (Relates to Figure 5)

Figure S2. (Relates to Figure 7 and Discussion)

Table S1 (Relates to Figure 2. The k_{cat}/K_M values used in the graph)

Table S2 (Relates to Figure 6. The list of the proteins tested in the Cdk1 substrate screen and relative k_{cat}/K_M values obtained)

Table S3 (Plasmid constructs used in this study)

Table S4 (Yeast strains used in this study)

Supplemental Discussion

Other cyclin-Cdk1 complexes

Supplemental Experimental Procedures

Plasmid constructs and yeast strains used in the study

Protein purification

Kinase assays

Western blotting

Peptide labeling with iTRAQ 4-plex reagents

Supplemental References

Figure S1

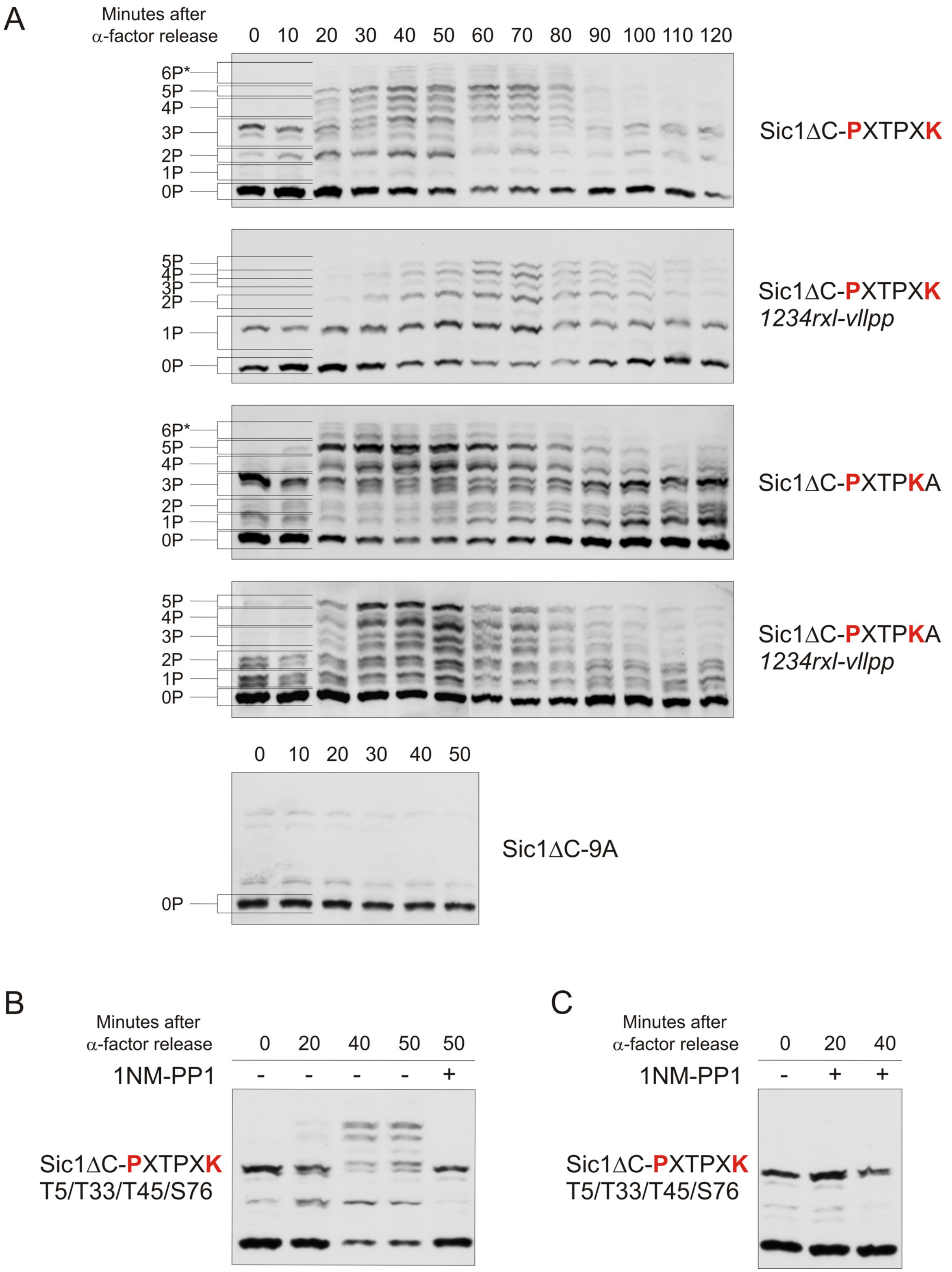
A
Minutes after
α-factor release
0 10 20 30 40 50 60 70 80 90 100 110 120
6P*
5P
4P
3P
2P
1P
0P
Sic1ΔC-PXTPXK
Sic1ΔC-PXTPXK
1234rxl-vllpp
Sic1ΔC-PXTPKA
Sic1ΔC-PXTPKA
1234rxl-vllpp
0 10 20 30 40 50
Sic1ΔC-9A
B
Minutes after
α-factor release
0 20 40 50 50
1NM-PP1
- - - - +
Sic1ΔC-PXTPXK
T5/T33/T45/S76
C
Minutes after
α-factor release
0 20 40
1NM-PP1
- + +
Sic1ΔC-PXTPXK
T5/T33/T45/S76

Figure S1 (continued)

D

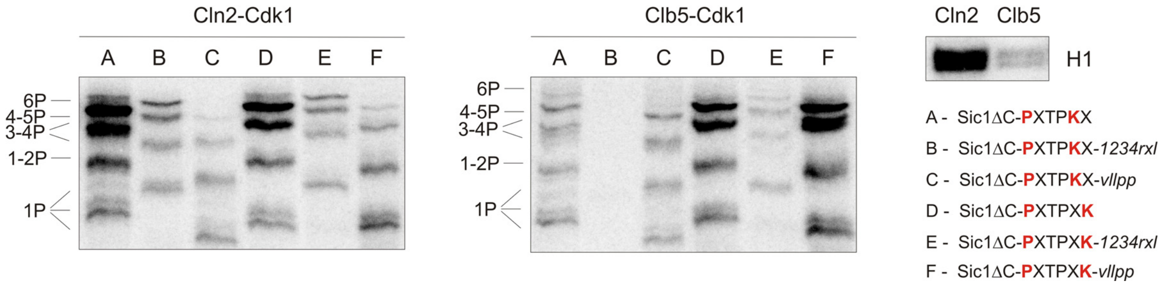

Figure S1 (related to Figure 5)

(A) Phos-Tag SDS-PAGE western blotting experiments using constitutively expressed Sic1ΔC-(T2)T5/T33/T45/T76 constructs bearing the Clb2-specific P-X-T-P-X-K or the Cln2-specific P-X-T-P-K-X substrate consensus motifs, and the same constructs containing both vllpp and 1234rxl mutations. Cells were released from α-factor arrest and the phosphorylation-dependent mobility shifts were followed as the cells progressed through the cell cycle. Sic1ΔC-3HA with all the Cdk1 consensus sites mutated to alanines (Sic1ΔC-9A) was included as a control. The signals were scanned using the GelDoc (GE) and the indicated phospho-bands were quantified to generate the plots shown in Figure 5A and B.

(B) We confirmed that the changing fraction of the phosphorylation shifts was Cdk1-dependent, using a yeast strain with an analog-sensitive Cdk1 allele (as-Cdk1, (Bishop et al., 2000)). The specific inhibitor 1NM-PP1 was added at the indicated time points after the shifts had already formed. The phosphoshifts present in G1 were not removed by the inhibitor and are probably caused by some other kinase. These bands were also included in the calculations of the steady-state phosphorylation levels.

(C) A similar experiment as in (B), except that the inhibitor was added immediately after the release of cells from the α-factor arrest.

(D) Cln2- and Clb5-dependent phosphorylation in vitro of Sic1ΔC-(T2)T5/T33/T45/T76 constructs and their docking site mutants (*vllpp* or *1234rxl*). Separation of the phosphorylated forms was performed using Phos-Tag SDS-PAGE followed by autoradiography.

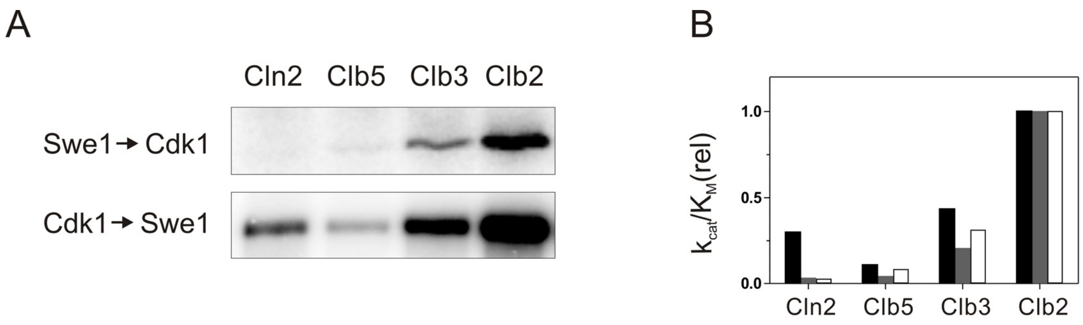

Figure S2 (related to Figure 7)

Inhibitory phosphorylation specificity of Swe1 towards Cdk1 is cyclin-specific and correlates with changes in the optimal consensus site specificity of Cdk1.

(A) Phosphorylation of different cyclin-Cdk1 complexes by purified Swe1 compared with phosphorylation of Swe1 by the same kinase complexes.

(B) The relative k_{cat}/K_M profile for Swe1-catalyzed phosphorylation of cyclin-Cdk1 complexes (open bars) compared with the k_{cat}/K_M profile for the cyclin-Cdk1-catalyzed phosphorylation of Swe1 (black bars) and of the model substrate PKTPKKAKKL (grey bars). The values for different cyclin-Cdk1 complexes are normalized relatively to the values obtained for Clb2-Cdk1.

Table S1

Specificity constants (k_{cat}/K_M) determined for four representative cyclin-Cdk1 complexes and for the hydrophobic patch mutants (hpm) of the three B-type cyclin-Cdk1 complexes using forms of T33-Sic1ΔC with varied amino acids around the phosphorylation site T33.

Substrate	k_{cat}/K_M, $\mu M^{-1}min^{-1}$						
	Cln2	Clb5	Clb3	Clb2	Clb5hpm	Clb3hpm	Clb2hpm
QK**TP**Q**K**PSQNL	3.21/ 0.92*	18.0	8.13	3.94	0.33	0.51	2.04
QK**TP**Q**K**PSQNL-*1234**rxl***	0.70/0.16	0.61	1.08	1.71	0.19	0.45	1.52
QK**TP**Q**A**PSQNL	1.00/0.11	1.38	0.27	0.26	0.02	0.04	0.16
**P**K**TP**Q**K**PSQNL	4.89/0.90	13.8	7.26	9.31	0.40	0.52	1.76
**P**K**TP**Q**A**PSQNL	1.49/0.29	0.30	0.20	0.35	0.03	0.04	0.09
QK**TPK**APSQNL	1.73/0.36	0.28	0.32	0.39	0.02	0.03	0.13
QK**TPR**APSQNL	3.02	2.74	0.73	0.39	-	-	-
**P**K**TPK**APSQNL	4.14/0.64	1.29	1.83	0.88	0.02	0.02	0.21
QK**TPKK**PSQNL	2.32/0.35	7.74	4.57	5.02	0.19	0.36	1.67
**P**K**TPKK**PSQNL	2.28/0.31	13.4	7.07	9.77	0.42	0.74	3.28
**P**K**TP**Q**KKKK**NL	2.74/0.41	24.4	31.7	39.0	1.11	4.22	17.3
**P**K**TP**Q**KKKK**NL *1234**rxl***	0.80/0.24	1.11	4.22	17.3	-	-	-
PK**TP**KKAKKLL**	4.97/1.26	50.0	58.0	24.1	1.35	4.85	12.35
QK**AP**QKPSQNL	0.02	0.04	0.04	0.10	0.06	0.09	0.07
QK**TA**QKPSQNL	0.05	0.10	0.16	0.19	0.02	0.02	0.04

* - Assay performed in the presence of LP peptide.
** - Cdk phosphorylation motif of Histone H1.

Table S2

Specificity constants (k_{cat}/K_M) for the phosphorylation of different substrates of Cdk1 determined with the four representative cyclin-Cdk1 complexes and the hydrophobic patch mutants (hpm) of the three B-type cyclin-Cdk1 complexes (relative values, not divided by [S]).

	k_{cat}/K_M (relative units)						
	Cln2	Clb5	Clb3	Clb2	Clb5hpm	Clb3hpm	Clb2hpm
Histone H1	1.394	0.605	2.237	4.900	0.427	1.957	4.287
ACE2	0.025	0.004	0.029	0.069	-	-	-
ASH1	0.422	0.111	0.697	0.462	0.052	0.134	0.389
BOP3	0.521	0.027	0.101	0.109	-	-	-
CDC6	0.084	2.898	2.268	0.078	0.138	0.350	1.688
CDH1	0.016	0.025	0.024	0.035	0.006	0.008	0.023
EXO84	0.084	0.019	0.031	0.074	0.006	0.014	0.062
FAR1	0.042	0.115	0.031	0.029	0.003	0.008	0.033
FIN1	1.800	23.02	10.96	12.43	3.651	7.488	14.08
FIR1	0.042	0.032	0.223	0.836	0.008	0.017	0.111
MSA1	0.157	0.024	0.043	0.034	-	-	-
NDD1	0.143	0.388	2.121	3.129	0.096	0.222	1.108
ORC2	0.073	0.405	0.132	0.018	0.012	0.028	0.067
ORC6	0.002	0.029	0.018	0.009	0.001	0.002	0.005
PDS1	0.249	0.008	0.015	0.049	0.002	0.005	0.035
PLM2	0.428	1.724	9.051	9.072	0.428	1.144	6.351
PXL1	0.031	0.031	0.139	0.571	0.015	0.033	0.244
RTT109	0.067	0.010	0.027	0.080	-	-	-
SLI15	0.422	0.025	0.141	0.109	0.004	0.032	0.060
STB1	2.011	0.191	0.622	0.878	-	-	-
SWI5	0.178	0.475	1.686	2.961	0.172	0.207	1.387
SWI6	0.004	0.118	0.105	0.412	0.058	0.075	0.481
TOS4	0.708	0.502	8.484	2.373	0.593	2.397	2.136
TOS8	2.830	0.069	0.276	0.254	-	-	-
WHI5	0.283	0.016	0.033	0.046	0.001	0.006	0.027
XBP1	0.200	0.020	0.065	0.103	0.007	0.013	0.084
YHP1	0.524	0.020	0.066	0.385	0.002	0.009	0.046
YML119w	0.048	0.029	0.134	0.130	-	-	-
YOX1	0.584	0.244	2.094	4.536	-	-	-

Table S3

Plasmid constructs used in this study.

Plasmid	Description
pMK0001-0052 variants	Sic1AA-T33QKTPQKPSQNL-ΔC-pET28a and mutated
pMK0063	ACE2-pET28a
pMK0064	ASH1-pET28a
pMK0065	BOP3-pET28a
pMK0066	CDC6-pET28b
pMK0067	EXO84-pET28a
pMK0068	FIN1-PET28a
pMK0069	FIR1-pET28a
pMK0070	NDD1-pET28a
pMK0071	ORC2-pET28a
pMK0072	ORC6-pRSAB1234
pMK0073	PDS1-pGEX-4T-1
pMK0074	PLM2-pET28a
pMK0075	PXL1-pET28a
pMK0076	RTT109-pET28a
pMK0077	SLI15-pET28a
pMK0078	STB1-pET28a
pMK0079	SWI5-pET28a
pMK0080	SWI6-pET28a
pMK0081	TOS4-pET28a
pMK0082	TOS8-pET28a
pMK0083	WHI5-pET28a
pMK0084	XBP1-pET28a
pMK0085	YML119W-pET28a
pMK0086	MSA1-pET28a
pMK0087	YHP1-pET28a
pMK0088	YOX1-pET28a
pMK0089	YPR174C-pET28a
pMK0090	Sic1AA-T33QKTPQKPSQNL-*1234rxl*-ΔC-pET28a
pMK0091	Sic1AA-ΔC-pET28a
pMK0092-0097	Sic1AP-T33-ΔC-pET28a and *rxl* variants
pMK0098-0103	Sic1AP-S76-ΔC-pET28a and rxl variants
pMK0104	Sic1AA-T33PKTPQAPSQNL-*1234rxl* -ΔC-pET28a
pMK0105	Sic1AA-T33QKTPKAPSQNL-*1234rxl* -ΔC-pET28a
pMK0106	Sic1AA-T33PKTPKAPSQNL-*1234rxl* -ΔC-pET28a
pMK0107	Sic1AA-T33PKTPQKKKKNL-*1234rxl* -ΔC-pET28a
pMK0114-0119	Sic1AA-T5-ΔC-pET28a and *rxl* variants
pMK0125	Sic1AP-T45-*1234rxl*-ΔC-pET28a
pMK0131	Sic1AP-S69-*1234rxl*-ΔC-pET28a
pMK0137	Sic1AP-S80-*1234rxl*-ΔC-pET28a
pMK0143	Sic1AP-T173-*1234rxl*-ΔC-pET28a
pMK0149	Sic1AP-S191-*1234rxl*-ΔC-pET28a
pMK0150	Sic1wt-ΔC-pET28a
pMK0184-0190	Sic1wt-T2(1-85)-pET28a and other truncated variants
pMK0191	Sic1wt-v*llpp*(136AAAAA140)-ΔC-pET28a
pMK0192	Sic1AP-PXTPKA-ΔC-pET28a
pMK0193	Sic1AP-PXTPKA-*1234rxl*-ΔC-pET28a
pMK0194	Sic1AP-PXTPKA-v*llpp*-ΔC-pET28a
pMK0195	Sic1AP-PXTPAK-ΔC-pET28a
pMK0196	Sic1AP-PXTPAK-*1234rxl*-ΔC-pET28a
pMK0197	Sic1AP-PXTPAK-v*llpp*-ΔC-pET28a

pMK0500	Sic1wt-3XHA-pRS413
pMK0502	Sic1wt-*1234rxl*-3XHA-pRS413
pMK0504	Sic1wt-*23rxl*-3XHA-pRS413
pMK0505	Sic1wt-*14rxl*-3XHA-pRS413
pMK0525	Sic1wt-*2rxl*-3XHA-pRS413
pMK0526	Sic1wt-*3rxl*-3XHA-pRS413
pMK0527	Sic1wt-v*llpp*-3XHA-pRS413
pMK0528	Sic1wt-*2rxl-vllpp*-3XHA-pRS413
pMK0529	Sic1wt-*3rxl-vllpp*-3XHA-pRS413
pMK0530	Sic1AP-PXTPKA-ΔC-3XHA-pRS315
pMK0531	Sic1AP-PXTPKA-*1234rxl-vllpp*-ΔC-3XHA-pRS315
pMK0532	Sic1AP-PXTPAK-ΔC-3XHA-pRS315
pMK0533	Sic1AP-PXTPAK-*1234rxl-vllpp*-ΔC-3XHA-pRS315
pMK0534	Sic1AP-ΔC-3XHA-pRS315
pMK0545	Sic1wt-*1234rxl-vllpp*-3XHA-pRS413

Table S4

Yeast strains used in this study.

Strain	Description
DOM0949	CLN2-3HA:HISMX6 bar1:hisG
DOM0962	CLB5-3HA:HISMX6 bar1:hisG
DOM0950	CLB3-3HA:HISMX6 bar1:hisG
DOM0948	CLB2-3HA:HISMX6 bar1:hisG
DOM0076	gal-CLB5-TAP pRSAB1234-URA3 bar1:HISG sic1d::LEU2
DOM0077	gal-CLB2-TAP pRSAB1234-URA3 bar1:HISG sic1d::LEU2
DOM0957	gal-CLB3-TAP pRSAB1234-URA3 bar1:HISG sic1d::LEU2
DOM0963	gal-CLB5hpm-TAP pRSAB1234-URA3 bar1:HISG sic1d::LEU2
DOM0964	gal-CLB2hpm-TAP pRSAB1234-URA3 bar1:HISG sic1d::LEU2
DOM0958	gal-SWE1-TAP pRSAB1234-URA3
MK0168	gal-CLB3hpm-TAP pRSAB1234-URA3 bar1:HISG sic1d::LEU2
MK0169	gal-ORC6-TAP pRSAB1234-URA3 bar1:HISG
DMY305	gal-CLN2-3HA
DOM0030	cdc28::cdc28 as1 bar1:HISG
DOM0090	bar1:HISG
MK0260	DOM0030 [Sic1AP-PXTPAK-ΔC-3XHA-pRS315]
MK0261	DOM0030 [Sic1AP-ΔC-3XHA-pRS315
MK0262	DOM0090 [Sic1AP-PXTPKA-ΔC-3XHA-pRS315]
MK0263	DOM0090 [Sic1AP-*1234rxl-vllpp*-PXTPKA-ΔC-3XHA-pRS315]
MK0264	DOM0090 [Sic1AP-PXTPAK-ΔC-3XHA-pRS315]
MK0265	DOM0090 [Sic1AP-*1234rxl-vllpp*-PXTPAK-ΔC-3XHA-pRS315]
MK0266	cln2d::TRP1; bar1:HISG
MK0267	DOM0090 [Sic1wt-3XHA-pRS413]
MK0268	DOM0090 [Sic1wt-*2rxl*-3XHA-pRS413]
MK0269	DOM0090 [Sic1wt-*3rxl*-3XHA-pRS413]
MK0270	DOM0090 [Sic1wt-*23rxl*-3XHA-pRS413]
MK0271	DOM0090 [Sic1wt-*14rxl*-3XHA-pRS413]
MK0272	DOM0090 [Sic1wt-*1234rxl*-3XHA-pRS413]
MK0273	DOM0090 [Sic1wt-v*llpp*-3XHA-pRS413]
MK0274	MK0266 [Sic1wt-3XHA-pRS413]
MK0275	MK0266 [Sic1wt-*vllpp*-3XHA-pRS413]
MK0276	MK0266 [Sic1wt-*2rxl*-3XHA-pRS413]
MK0277	MK0266 [Sic1wt-*3rxl*-3XHA-pRS413]
MK0278	MK0266 [Sic1wt-*2rxl-vllpp*-3XHA-pRS413]
MK0279	MK0266 [Sic1wt-*3rxl-vllpp*-3XHA-pRS413]
MK0310	DOM0090 [Sic1wt-*1234rxl-vllpp*-3XHA-pRS413]

Supplemental Discussion

The four major cyclin-Cdk1 complexes used in our experiments seem sufficient for describing the general dynamic changes of Cdk1 specificity during the cell cycle, as the rest of the cyclins, Clb1 and Clb4 (Grandin and Reed, 1993) as well as Cln3 and Clb6 (Cross et al., 2002; Tyers et al., 1993) are less abundant and therefore minor contributors to the total concentration of activated Cdk1, and most likely would not yield significant changes to the general model. In addition, we found that similarly to Clb3-Cdk1, Clb4-Cdk1, the other G2 complex, also exhibited an intermediate K_M (210 μM) for the model substrate (data not shown). However, due to poor purification yields, this kinase was excluded from detailed analysis. Additionally, the other G1 complex Cln1-Cdk1, like the closely related Cln2-Cdk1 complex, showed low intrinsic activity towards the peptide substrate (K_M > 1 mM, data not shown).

Our findings have raised an intriguing question of how cyclins can change the specificity of the Cdk1 active site, so that its ability to bind and phosphorylate the consensus site motif is altered. These cyclin-specific effects could be due to specific conformational changes induced by cyclin in the Cdk1 molecule, resulting in altered accessibility and configuration of its active site. Interestingly, differential accessibility of the active site is also suggested by the differing ability of Swe1 to phosphorylate different cyclin-Cdk1 complexes at the active site-gating Y19 residue. The other gradually changing feature, the relative differences in RXL-dependent docking mechanisms among the B-type cyclin-Cdk1s, could also be due to different specific conformational changes induced by cyclins, resulting in changes in optimal distance requirements or structural hindrances between the RXL-hp docking site and the active site.

Supplemental Experimental Procedures

Plasmid constructs and yeast strains

For tandem affinity purification (TAP) of cyclin-Cdk1 complexes, the cyclin genes were cloned into the 2 micron vector pRSAB1234 containing a *GAL1* promoter and the C-terminal TAP-tag. To purify different 6His-T33-Sic1ΔC forms for kinase assays, the C-terminally truncated fragment of Sic1 (with a stop codon inserted after amino acid 215) was cloned into the NheI/BamHI site of the bacterial expression vector pET28a (Invitrogen). Mutagenesis of cyclins or substrates was performed using single-stranded pRSAB1234 or pET28a vector constructs as templates. The triple mutants in the hydrophobic patch (hpm) of Clb5 and Clb2 were described previously (Loog and Morgan, 2005), and the hpm of Clb3 (Phe-201, Leu-205 and Trp-208 mutated to alanines) was designed according to sequence homology with other B-type cyclins. In T33-Sic1ΔC constructs, the other Cdk sites bearing the consensus motif S/T-P were mutated to Ala-Ala in order to prevent any possible (pseudo)substrate competition. The other Cdk substrates were cloned as full-length forms into bacterial expression vectors pET28a or pGEX-4T-1. Cdh1 was purified using the baculovirus system and was a kind gift from Monica Rodrigo-Brenni (UCSF). For purification of 3HA-Cln2-Cdk1, a yeast strain DMY305 (a kind gift from Dr Doug Kellogg, UCSC) with the galactose promoter introduced along with the N-terminal 3HA-tag in the chromosomal locus of the *CLN2* gene was used. The vector construct pCKS1 for bacterial expression of Cks1 was a kind gift from Dr. Adam Rudner. The C-terminally TAP-tagged tyrosine kinase Swe1 was cloned and purified using the 2 micron vector pRSAB1234 analogously to the cyclin-Cdk1 complexes. For C-terminal tagging of cyclin genes in their chromosomal loci, the Pringle method was applied (Longtine et al., 1998). Lists of plasmid vectors and yeast strains used in this study are presented in Tables S3 and S4, respectively.

Protein purification

The TAP method was applied for purification of cyclin-Cdk1 complexes and Swe1 as described previously for Clb5-TAP-Cdk1 and Clb2-TAP-Cdk1 (Puig et al., 2001; Ubersax et al., 2003). For purification of 3HA-Cln2-Cdk1, the yeast strain DMY305 was induced with 2% galactose for 1 hour, and the 3HA-Cln2-Cdk1 complex was purified according to published protocols (McCusker et al., 2007), exploiting immunoaffinity chromatography with a rabbit polyclonal antibody raised against the synthetic 2HA peptide (purchased from Labas, Estonia). We confirmed that the specificity of the Cln2-Cdk1 complex was independent of the nature of the affinity tag used, since Cln2-3HA showed

similar substrate specificity as the Cln2-Cdk1 version purified using a TAP-tag. N-terminally 6His-tagged recombinant T33-Sic1ΔC constructs and substrates were cloned into the pET28a vector and purified by standard cobalt affinity chromatography and elution with 200 mM imidazole. 6His-Fin1 was purified as described previously (Woodbury and Morgan, 2007). Substrates cloned into the pGEX-4T-1 were purified on glutathione agarose columns using 5 mM reduced glutathione for elution. Cks1 was purified as described previously (Reynard et al., 2000). The optimal working concentration for purified Cks1 was taken as 500 nM based on optimization performed for cyclin-Cdk1 preparations and T33-Sic1ΔC as a substrate.

Kinase assays

For the quantitative phosphorylation assays of T33-Sic1ΔC constructs and recombinant substrates, substrate concentrations were kept in the range of 0.5-2 μM (in the linear [S] vs v_0 range, several-fold below the estimated K_M value), and the initial velocity conditions were defined as an initial substrate turnover ranging up to 10% of the total turnover. The latter was estimated by a long-term incubation with excess amounts of cyclin-Cdk1 and ^{32}P-γ-ATP in the standard reaction mixture given below. About 1-10 nM of purified kinase complex was used, reaction aliquots were taken at two or more time points, and the reaction was stopped by SDS-PAGE sample buffer. The relative k_{cat}/K_M values for the substrates were calculated as the ratio of v_0/[S], which was determined from at least two independent experiments. The basal composition of the assay mixture contained 50 mM Hepes, pH 7.4, 100 mM NaCl, 0.1% NP-40, 20 mM imidazole, 0.1 mg/ml 2HA peptide, 2% glycerol, 2 mM EGTA, 0.2 mg/ml BSA, 80 μg/ml Cks1, and 500 μM ATP (with added ^{32}P-γ-ATP (Perkin Elmer)). About 1-10 nM of purified kinase complex was used, reaction aliquots were taken at two or more time points, and the reaction was stopped by SDS-PAGE sample buffer. For the steady-state peptide kinetics of the Histone peptide PKTPKKAKKL, a similar assay composition was used as for protein substrates and the standard phosphocellulose method was applied for the quantification of the phosphorylated substrate (Loog and Morgan, 2005). Synthetic peptides as competitor agents were used in 4 mM final concentrations.

Phosphorylation of Cdk1 by purified Swe1 was performed in the standard kinase assay mixture, except that 10 μM ATP was used to amplify the radioactive signal. The Swe1 concentration was optimized so that it was possible to take assay points below 10% of Cdk1 Y19 phosphorylation turnover, and the k_{cat}/K_M values for Swe1 as the enzyme and for cyclin-Cdk1 as the substrate were estimated as described above for the Cdk1 substrates. For the Cdk1 active site titration

experiments, similar conditions were used, except that more than a 10-fold higher concentration of purified Swe1 was included (Figure 1D).

Western blotting

The antibody used for the western blotting of 3HA-tagged proteins was HA.11 Clone 16B12 from Covance, USA. The antibody for the detection of Cdk1 was Cdc28 (yC-20) sc-6709 and the antibody to detect the zz-domain of the TAP-tag was cMyc (A-14) sc-789, both from Santa Cruz Biotechnology, USA. The antibody for the detection of inhibitory phosphorylation at Y19 in Cdk1 was Phospho-cdc2 (Y15) from Cell Signaling Technology and that for the detection of activating phosphorylation of Cdk1 at T169 was a kind gift from Dr Philip Kaldis (IMCB). In case of the time course experiments, the cells were lysed in lysis buffer containing 8M urea, 2M thiourea, 20 mM Tris pH7.4, 4% CHAPS, 1% DTT, 50 mM NaF, 89 mM β-gycerophosphate, 1 mM Na_3VO_4.The protein concentration of the samples was determined using the Bradford method.

Peptide labeling with iTRAQ 4-plex reagents

Purified cyclin-Cdk1 complexes were separated by 1D SDS-PAGE and the gel stained with Colloidal Coomassie blue. Protein bands containing Cdk1 were excised from the gel and the protein within them was submitted to reduction, alkylation and tryptic digestion in 100 mM triethyl ammonium bicarbonate (TEAB) (Applied Biosystems).

Dried tryptic peptides were dissolved in 100 mM triethyl ammonium bicarbonate (TEAB) containing the iTRAQ reagents (114, 115, 116 or 117) (Applied Biosystems) and final concentration of 25% (v/v) ethanol. The samples were incubated at room temperature for 1 h and the excess of reagent was quenched by adding MQ water. After 30 minutes incubation at room temperature samples were pooled together in a 1:1:1:1 ratio (v/v) and dried down in a vacuum centrifuge and purified on C18 StageTips (Rappsilber et al., 2007)

Peptides were separated by reverse-phase chromatography using an Agilent 1200 series nanoflow system (Agilent Technologies) connected to a LTQ Orbitrap classic mass-spectrometer (Thermo Electron, Bremen, Germany) equipped with a nanoelectrospray ion source (Proxeon, Odense, Denmark), essentially as described in (Pulk et al., 2010). Data acquisition and analysis was done as described in (Kocher et al., 2009), with slight modifications. Peak lists were extracted from .raw files with Proteome Discoverer 1.1 and searched with Mascot 2.3 (www.matrixscience.com) against yeast database complemented with common contaminant sequences such as trypsin,

keratins etc. Peptides were identified based on CID MS/MS scans and reporter ions from HCD scans were extracted manually from corresponding .raw files.

Supplemental References

Cross, F.R., Archambault, V., Miller, M., and Klovstad, M. (2002). Testing a mathematical model of the yeast cell cycle. Mol. Biol. Cell *13,* 52-70.

Grandin, N., and Reed, S.I. (1993). Differential function and expression of Saccharomyces cerevisiae B-type cyclins in mitosis and meiosis. Mol. Cell. Biol. *13,* 2113-2125.

Kocher, T., Pichler, P., Schutzbier, M., Stingl, C., Kaul, A., Teucher, N., Hasenfuss, G., Penninger, J.M., and Mechtler, K. (2009). High precision quantitative proteomics using iTRAQ on an LTQ Orbitrap: a new mass spectrometric method combining the benefits of all. J. Proteome Res. *8,* 4743-4752.

Longtine, M.S., McKenzie, A.,3rd, Demarini, D.J., Shah, N.G., Wach, A., Brachat, A., Philippsen, P., and Pringle, J.R. (1998). Additional modules for versatile and economical PCR-based gene deletion and modification in Saccharomyces cerevisiae. Yeast *14,* 953-961.

Loog, M., and Morgan, D.O. (2005). Cyclin specificity in the phosphorylation of cyclin-dependent kinase substrates. Nature *434,* 104-108.

McCusker, D., Denison, C., Anderson, S., Egelhofer, T.A., Yates, J.R.,3rd, Gygi, S.P., and Kellogg, D.R. (2007). Cdk1 coordinates cell-surface growth with the cell cycle. Nat. Cell Biol. *9,* 506-515.

Puig, O., Caspary, F., Rigaut, G., Rutz, B., Bouveret, E., Bragado-Nilsson, E., Wilm, M., and Seraphin, B. (2001). The tandem affinity purification (TAP) method: a general procedure of protein complex purification. Methods *24,* 218-229.

Pulk, A., Liiv, A., Peil, L., Maivali, U., Nierhaus, K., and Remme, J. (2010). Ribosome reactivation by replacement of damaged proteins. Mol. Microbiol. *75,* 801-814.

Rappsilber, J., Mann, M., and Ishihama, Y. (2007). Protocol for micro-purification, enrichment, pre-fractionation and storage of peptides for proteomics using StageTips. Nat. Protoc. *2,* 1896-1906.

Reynard, G.J., Reynolds, W., Verma, R., and Deshaies, R.J. (2000). Cks1 is required for G(1) cyclin-cyclin-dependent kinase activity in budding yeast. Mol. Cell. Biol. *20,* 5858-5864.

Tyers, M., Tokiwa, G., and Futcher, B. (1993). Comparison of the Saccharomyces cerevisiae G1 cyclins: Cln3 may be an upstream activator of Cln1, Cln2 and other cyclins. EMBO J. *12,* 1955-1968.

Ubersax, J.A., Woodbury, E.L., Quang, P.N., Paraz, M., Blethrow, J.D., Shah, K., Shokat, K.M., and Morgan, D.O. (2003). Targets of the cyclin-dependent kinase Cdk1. Nature *425,* 859-864.

Woodbury, E.L., and Morgan, D.O. (2007). Cdk and APC activities limit the spindle-stabilizing function of Fin1 to anaphase. Nat. Cell Biol. *9,* 106-112.
